# Pulmonary Mucormycosis in a Patient with Systemic Lupus Erythematosus: A Diagnostic and Treatment Challenge

**DOI:** 10.1155/2015/478789

**Published:** 2015-06-22

**Authors:** Hung-Chang Hung, Gang-Yu Shen, Shiuan-Chih Chen, Kai-Jieh Yeo, Shih-Ming Tsao, Meng-Chih Lee, Yuan-Ti Lee

**Affiliations:** ^1^Division of Gastroenterology, Department of Internal Medicine, Nantou Hospital, Ministry of Health and Welfare, No. 478, Fuxing Road, Nantou City, Nantou County 540, Taiwan; ^2^Division of Pulmonary and Critical Care Medicine, Department of Internal Medicine, Chiu General Hospital, No. 137, Chenggong First Road, Lingya District, Kaohsiung 802, Taiwan; ^3^Department of Family and Community Medicine, Chung Shan Medical University Hospital, No. 110, Section 1, Jianguo North Road, Taichung 402, Taiwan; ^4^School of Medicine, Chung Shan Medical University, No. 110, Section 1, Jianguo North Road, Taichung 402, Taiwan; ^5^Institute of Medicine and School of Medicine, Chung Shan Medical University, No. 110, Section 1, Jianguo North Road, Taichung 402, Taiwan; ^6^Division of Allergy, Immunology, Rheumatology, Department of Internal Medicine, Chung Shan Medical University Hospital, No. 110, Section 1, Jianguo North Road, Taichung 402, Taiwan; ^7^Division of Infectious Diseases, Department of Internal Medicine, Chung Shan Medical University Hospital, No. 110, Section 1, Jianguo North Road, Taichung 402, Taiwan; ^8^Department of Family Medicine, Taichung Hospital, Ministry of Health and Welfare, No. 199, Section 1, Sanmin Road, Taichung 403, Taiwan

## Abstract

Pulmonary mucormycosis is commonly encountered in patients with diabetic ketoacidosis, hematologic malignancies, neutropenia, organ or hematopoietic stem cell transplantation, and malignancy, but it rarely occurs in high-risk patients with systemic lupus erythematosus (SLE). We present the case of a 40-year-old SLE female with fulminant pneumonia after remission of nephritis treated with rituximab, who developed severe pulmonary mucormycosis that led to her rapid death from acute respiratory failure and acute respiratory distress syndrome. Pulmonary mucormycosis has a high mortality rate. However, with early diagnosis and antifungal therapy with lipid formulation-liposomal amphotericin B and surgical removal of the infected area, the outcome can be improved.

## 1. Introduction

The most common invasive fatal opportunistic diseases (*Rhizopus* 47% and* Mucor* 18%) are of the order Mucorales. These include* Rhizopus*,* Mucor*,* Cunninghamella*,* Apophysomyces*,* Absidia*,* Saksenaea*,* Rhizomucor*,* Cokeromyces*, and* Syncephalastrum* species. Infections caused by both mucormycosis and entomophthoramycosis were previously called zygomycosis. The class Zygomycetes has disappeared from current taxonomy. The infections caused by the order Mucorales are now termed mucormycosis [1–3].

There are several potential contributing factors to mucormycosis including poorly controlled diabetes mellitus (both types 1 and 2), diabetic ketoacidosis, metabolic acidosis, persistent neutropenia, high-dose glucocorticoid therapy, trauma or burns, and iron overload due to chelation therapy with deferoxamine in patients on dialysis or chronically transfusion dependent [1, 4]. Invasive fungal infection is rarely encountered among SLE patients. Of these, the most common pathogens are cryptococcosis, candidiasis, and aspergillosis [5]. The mortality rate of mucormycosis ranges from 5 to 95% and that of pulmonary mucormycosis is 50–95% [1, 3, 6–8]. There are very few reports of mucormycosis among SLE patients and the mortality could be over 50% [9–11]. The clinical features of mucormycosis include rhinocerebral (39–66%), pulmonary (16–24%), central nervous system (9%), and gastrointestinal (7%) and local cutaneous involvement (10–19%) forms [3, 7].

This paper describes a patient receiving rituximab for SLE nephritis after remission that presented with severe pneumonia which was complicated by acute respiratory distress syndrome because of invasive mucormycosis.

## 2. Case Report

**Figure 1:**
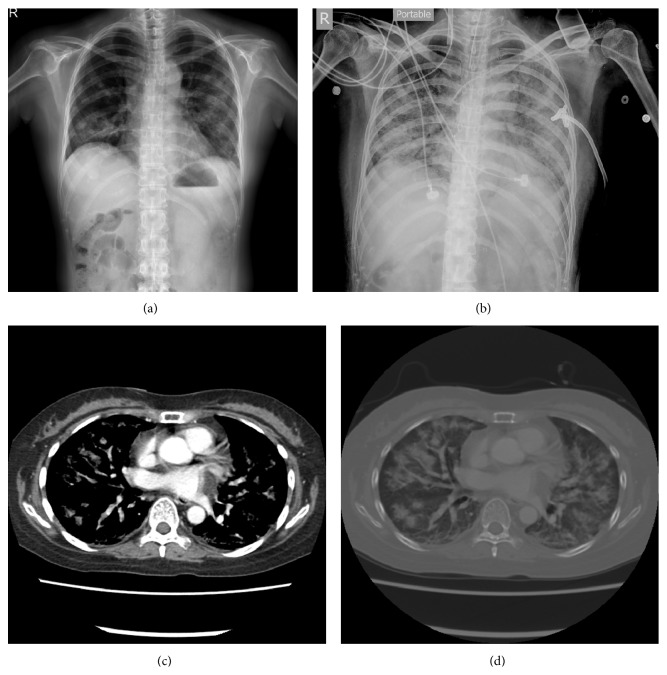
(a) Admission anteroposterior chest radiography shows multilobar solitary nodules and consolidation. (b) Anteroposterior chest radiography on hospital day 10 shows diffuse consolidation of bilateral lungs and pneumothorax with subcutaneous emphysema in soft tissues. (c) Chest CT scan (contrast) shows diffuse irregular rim of consolidation and multiple solitary nodules of bilateral lungs. (d) Chest CT scan (lung window) shows diffuse consolidation with multiple air lucencies and solitary nodules of bilateral lungs.

**Figure 2:**
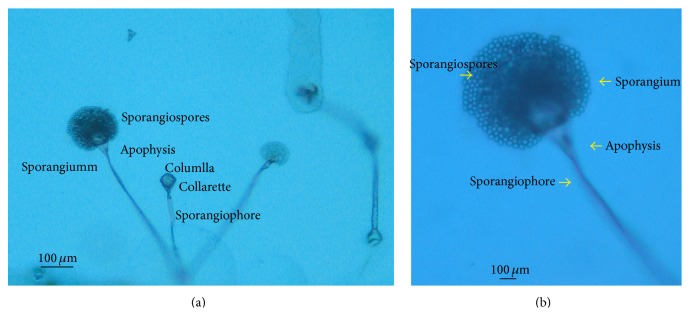
Microscopic features of the* Rhizopus* species isolated from bronchoalveolar lavage culture of the patient. (a) Sporangiophores are long and nondichotomous, usually terminating in large globose sporangia (100×). (b) Sporangiophore with sporangium contains numerous sporangiospores (400×).

A 40-year-old female patient arrived at the Emergency Department of the Chung Shang Medical University Hospital, Taichung, Taiwan, because she had a fever, chills, and shortness of breath for a period of one week. Her past medical history was SLE nephritis for which she was put on a monthly dose of rituximab target therapy for the previous three months at a medical center hospital in Taipei, Taiwan. She did not have any pulmonary problems prior to this episode. On physical examination, the patient had a body temperature of 38.8°C (101.8°F), a heart rate of 140/min, and respirations at 24/min. Blood pressure was 142/89 mm Hg and oxygen saturation on room air was 90%. A complete blood count revealed hemoglobin levels of 11.4 g/dL, white cell counts of 2,010 cells/*μ*L (myeloblasts 6%, metamyelocytes 4%, bands 13%, neutrophils 69%, and leukocytes 5%), and platelet counts of 145,000 cells/*μ*L. Blood biochemistry revealed lactate dehydrogenase (LDH) 1097 U/L; procalcitonin (PCT) 0.26 ng/mL; C-reactive protein (CRP) 11.1 mg/dL (normal range: <0.4). On hospital day 7, a lymphocytes cell laboratory testing through flow cytometry revealed white cell counts of 4,090 cells/*μ*L (myeloblasts 4%, metamyelocytes 8%, bands 7%, neutrophils 78%, and leukocytes 3%), absolute lymphocyte count 353 cells/*μ*L (9%), absolute CD19 B lymphocyte 64 cells/*μ*L (18.1%), absolute CD4 T-cell count 177 cells/*μ*L, and absolute CD8 T-cell count 127 cells/*μ*L. A chest X-ray after admission revealed the presence of bilateral infiltration and poorly defined nodular, cavitary opacities in both lower lungs (Figures 1(a) and 1(b)) and a clinical diagnosis of pneumonia was made. After admission, she was treated with piperacillin/tazobactam and azithromycin, but the patient experienced rapid progression to acute respiration distress. Fluconazole and methylprednisolone were put on hospital day two. On the third day of hospitalization, she developed septic shock and acute respiratory failure and required urgent ventilation and extracorporeal membrane oxygenation (ECMO). Piperacillin/tazobactam, azithromycin, fluconazole, and methylprednisolone were discontinued and meropenem, sulfamethoxazole/trimethoprim for pneumocystis pneumonia, and micafungin (3.5 mg/kg/day) for invasive fungal infection were started at that time. Three days after admission, bronchoalveolar lavage (BAL) showed blood tinged mucous fluid and numerous macrophages and a bit of Gram-negative bacilli and yeast on a Gram stain. A chest computed tomography (CT) image reveals a minimal amount of pleural effusion and poorly defined nodular, cavitary opacities and ground-glass opacities in both lower lungs (Figures 1(c) and 1(d)). Microbiological and serological studies for* Legionella pneumophila*, Human Immunodeficiency Virus-1, Epstein-Barr virus,* Cytomegalovirus*,* Cryptococcus neoformans*,* Streptococcus pneumoniae*,* Chlamydia pneumoniae*,* Mycoplasma pneumonia,* and tuberculosis were all negative. Three sets of blood cultures drawn at admission and bacteria cultures of sputum were sterile after seven days of incubation. The patient's condition deteriorated in the intensive care unit. On hospital day 11, the patient was declared dead. Cultures from BAL yielded* Rhizopus* species on hospital day 13 (Figures 2(a) and 2(b)). By now there was a clinical diagnosis of pulmonary infection with mucormycosis.

## 3. Discussion

There has been a marked increase in the incidence of opportunistic fungal infections worldwide and an even more pronounced increase with mucormycosis [3, 8]. The clinical manifestations of mucormycosis are broad and depend on the patient's immune status and underlying conditions [3]. There has been an increase in the number of reports of mucormycosis in patients with SLE [11]. All patients with SLE that are immunocompromised involve intrinsic defects in immune function and hypogammaglobulinemia and as such are typically receiving immunosuppressive medicine anti-CD20 monoclonal antibody (mAb) rituximab and glucocorticoid therapy for control of SLE nephritis. Thus, such patients, including the patient described in this case report, are at high risk of invasive fungal infection.

Long-term corticosteroid therapy enhances a patient's susceptibility to mucormycosis by causing defects in macrophages and neutrophils [1, 12]. The adverse events of mAb include infusion-related acute anaphylaxis, serum sickness, infections, dermatitis, cancer, autoimmune disease, and cardiotoxicity [13]. Types of infectious complications related to rituximab include bacterial infections, viral reactivation, mycobacterial infections, fungal infections, and protozoal infections [13]. Invasive aspergillosis and candidiasis have been reported in high-risk patients; however, pulmonary mucormycosis related to rituximab treatment has not been reported before [13, 14]. In this case, the use of rituximab might have affected CD4 T-cell dysfunction and was suspected as contributing to pulmonary mucormycosis [14].

At first, she suffered from dyspnea and fever intermittently for 7 days. A chest X-ray revealed both cavitary lower lungs and opacity infiltration. Hence, the tentative diagnosis was SLE relapse and a pulmonary infection. The high level of CRP was probably indicative of a bacterial or fungal infection. She was initially treated with a broad-spectrum antimicrobial agent and preemptive antifungal agent such as fluconazole and micafungin. The clinical course of pneumonia was so fulminant that despite using ECMO in the ICU the patient still died. The sputum culture showed* Rhizopus* species after her death.

Pulmonary mucormycosis develops after inhalation of fungal sporangiospores, by spreading from hematogenous or lymphatic system to the lungs or angioinvasion causing necrosis and infarction of the affected tissues [8]. Pulmonary mucormycosis can present as mild to severe such as fever unresponsive to antibiotics, dyspnea, cough, pleuritic chest pain, and hemoptysis [7]. It may invade organs adjacent to the lungs, such as the mediastinum, pericardium, and chest wall, or it may disseminate systemically. Invasion of the large mediastinal vessels can lead to massive hemoptysis, which has occasionally been fatal [1]. The signs of pulmonary mucormycosis on chest images are nonspecific and indistinguishable from those of pulmonary aspergillosis [8]. Findings include consolidation, nodules, masses, cavities, pleural effusion, atelectasis, posterior tracheal band thickening, and hilar or mediastinal lymphadenopathy. Chest radiograph can even be normal [7]. When reading chest CT images, the air crescent sign (a thin rim of air between the necrotic lung and the surrounding parenchyma) and the halo sign (consolidation with a rim of surrounding) suggest the presence of an invasive fungal infection [7, 8]. The reverse halo sign may be an early indicator of pulmonary mucormycosis. These images are also able to detect lesions [8]. But these cannot distinguish mucormycosis from aspergillosis.

Direct examination of sputum, paranasal sinus secretions, or BAL fluid is frequently nondiagnostic, but isolation of Mucorales organisms in such specimens from a susceptible host with pneumonia or rhinocerebral infection should be considered a strong indicator of mucormycosis [8]. Direct histological examination of a tissue biopsy remains the gold standard for diagnosis [8, 12]. In the patient, the clinical diagnosis of pulmonary mucormycosis was based on the clinical manifestations and result of a BAL culture. An autopsy was not performed. It is not possible to affirm that the patient undoubtedly had pulmonary mucormycosis by direct histological examination. Newer molecular diagnostic techniques, such as polymerase chain reaction (PCR), might permit a more rapid diagnosis and provide increasingly sensitive results, potentially improving the outcome in these critical patients [8, 12].

This patient developed ARDS and was quickly treated with antimicrobials. We believe that a delay in appropriate antifungal treatment probably contributed to the poor outcome. It is important to distinguish mucormycosis from aspergillosis because the treatments can differ and because early appropriate antifungal therapy of mucormycosis may improve outcomes [8, 12]. Effective management options for pulmonary mucormycosis consist of early diagnosis, appropriate antifungal therapy, aggressive surgical debridement, and correction of the causes of immunosuppression [6]. This patient initially received fluconazole and micafungin as a preemptive treatment. Azoles and echinocandin antifungal drugs are not effective against mucormycosis, although they are in the treatment of aspergillosis. Amphotericin B or its lipid formulations of amphotericin B are recommended as effective agents against invasive mucormycosis [3, 8]. More recently, posaconazole is efficacious when used in either mono-, combination-, or salvage-therapy in the treatment of mucormycosis [6, 7].

In summary, mucormycosis occurs most frequently in patients with diabetes mellitus, neutropenia coupled with hematological diseases, and organ transplants and patients receiving immunosuppressive medication [1]. Pulmonary mucormycosis in SLE patients is relatively rare and is associated with elevated morbidity and mortality. More rapid and accurate diagnostic methods and the availability of more effective antifungal drugs may help improve the prognosis of cases involving mucormycosis in the near future.
